# Supplementary material for: Precise modulation of BRG1 levels reveals features of mSWI/SNF dosage sensitivity
Source: Nat Genet. 2025 Aug 22;57(9):2250–63. doi: 10.1038/s41588-025-02305-z (PMC12425804; doi:10.1038/s41588-025-02305-z)
Supplement: Supplementary file 1 — Reporting Summary [file 41588_2025_2305_MOESM1_ESM.pdf]

nature portfolio

Corresponding author(s): Yi Zhang

Last updated by author(s): 2025/07/16

Reporting Summary

Nature Portfolio wishes to improve the reproducibility of the work that we publish. This form provides structure for consistency and transparency in reporting. For further information on Nature Portfolio policies, see our Editorial Policies and the Editorial Policy Checklist.

Statistics

For all statistical analyses, confirm that the following items are present in the figure legend, table legend, main text, or Methods section.

n/a	Confirmed	
☐	☒	The exact sample size (*n*) for each experimental group/condition, given as a discrete number and unit of measurement
☐	☒	A statement on whether measurements were taken from distinct samples or whether the same sample was measured repeatedly
☐	☒	The statistical test(s) used AND whether they are one- or two-sided<br>*Only common tests should be described solely by name; describe more complex techniques in the Methods section.*
☒	☐	A description of all covariates tested
☐	☒	A description of any assumptions or corrections, such as tests of normality and adjustment for multiple comparisons
☐	☒	A full description of the statistical parameters including central tendency (e.g. means) or other basic estimates (e.g. regression coefficient) AND variation (e.g. standard deviation) or associated estimates of uncertainty (e.g. confidence intervals)
☐	☒	For null hypothesis testing, the test statistic (e.g. *F*, *t*, *r*) with confidence intervals, effect sizes, degrees of freedom and *P* value noted<br>*Give P values as exact values whenever suitable.*
☒	☐	For Bayesian analysis, information on the choice of priors and Markov chain Monte Carlo settings
☒	☐	For hierarchical and complex designs, identification of the appropriate level for tests and full reporting of outcomes
☒	☐	Estimates of effect sizes (e.g. Cohen's *d*, Pearson's *r*), indicating how they were calculated

Our web collection on statistics for biologists contains articles on many of the points above.

Software and code

Policy information about availability of computer code

Data collection	No software was used
Data analysis	The codes for analyzing the sequencing data are available at GitHub: https://github.com/YiZhang-lab/Brg1_dTag and at Zenodo: https://doi.org/10.5281/zenodo.15951452.

For manuscripts utilizing custom algorithms or software that are central to the research but not yet described in published literature, software must be made available to editors and reviewers. We strongly encourage code deposition in a community repository (e.g. GitHub). See the Nature Portfolio guidelines for submitting code & software for further information.

Data

Policy information about availability of data

All manuscripts must include a data availability statement. This statement should provide the following information, where applicable:

- Accession codes, unique identifiers, or web links for publicly available datasets
- A description of any restrictions on data availability
- For clinical datasets or third party data, please ensure that the statement adheres to our policy

The sequencing data reported in this study are available at Gene Expression Omnibus: GSE274469 and GSE294015. ChromBPNet models were uploaded to figshare: https://doi.org/10.6084/m9.figshare.28705430. Source data are provided with this paper. All other data supporting the findings of this study are available from the corresponding author on reasonable request.

Research involving human participants, their data, or biological material

Policy information about studies with human participants or human data. See also policy information about sex, gender (identity/presentation), and sexual orientation and race, ethnicity and racism.

Reporting on sex and gender	N/A
Reporting on race, ethnicity, or other socially relevant groupings	N/A
Population characteristics	N/A
Recruitment	N/A
Ethics oversight	N/A

Note that full information on the approval of the study protocol must also be provided in the manuscript.

Field-specific reporting

Please select the one below that is the best fit for your research. If you are not sure, read the appropriate sections before making your selection.

☒ Life sciences ☐ Behavioural & social sciences ☐ Ecological, evolutionary & environmental sciences

For a reference copy of the document with all sections, see nature.com/documents/nr-reporting-summary-flat.pdf

Life sciences study design

All studies must disclose on these points even when the disclosure is negative.

Sample size	No statistical method was used to predetermine sample size but our sample sizes are similar to those reported in previous publications (PMID: 39747581, 33558760, 33558757). For publicly available datasets used in the study, we analyze the relevant experimental system (mESCs, BEAS-2B).
Data exclusions	Drosophila DNA for ATAC-seq and spike-in yeast DNA for CUT&RUN are excluded from analysis due to substantial variation in spike-in read counts across samples, which is possibly caused by inconsistent spike-in DNA amounts. Three data points were excluded from the cell proliferation assay because their values exceeded the detectable range or were suspected to result from uneven cell seeding.
Replication	All experiments were performed 2-6 biological replicates as indicated in manuscript. All attempts are replications were successful, all samples are included in manuscript.
Randomization	Randomization was not considered in this cell culture-based study. Comparison were done between treated and control cells.
Blinding	Blinding was not considered for this study. All cells were grown in identical culture condition (+/- treatment). No subjective measurements were applied.

Reporting for specific materials, systems and methods

We require information from authors about some types of materials, experimental systems and methods used in many studies. Here, indicate whether each material, system or method listed is relevant to your study. If you are not sure if a list item applies to your research, read the appropriate section before selecting a response.

Materials & experimental systems

n/a	Involved in the study	
☐	☒	Antibodies
☐	☒	Eukaryotic cell lines
☒	☐	Palaeontology and archaeology
☐	☒	Animals and other organisms
☒	☐	Clinical data
☒	☐	Dual use research of concern
☒	☐	Plants

Methods

n/a	Involved in the study	
☒	☐	ChIP-seq
☒	☐	Flow cytometry
☒	☐	MRI-based neuroimaging

Antibodies

Antibodies used

BRG1 rabbit monoclonal (Abcam, ab110641, Lot# GR3375498-11)
HA-Tag rabbit monoclonal (Cell Signaling Technology, 3724S, Lot# 11)
HA-Tag mouse monoclonal (Cell Signaling Technology, 2367S, Lot# 5)
ARID1A mouse monoclonal (Novus Biologicals, NBP2-61623, Lot# MAB-03564)
PBRM1 rabbit polyclonal (Bethyl Laboratories, A301-591A, Lot# 5)
BRD9 rabbit polyclonal (Bethyl Laboratories, A303-781A, Lot# 3)
HDAC1 mouse monoclonal (Novus Biologicals, NBP2-52937, Lot# MAB-03647)
SNF2H rabbit polyclonal (Abcam, ab72499, Lot# GR255705-60)
Rhodamine-conjugated Tubulin (Bio-Rad, 12004165, Lot# 64512248)
Normal Rabbit IgG (Cell Signaling Technology, 2729S, Lot# 11)
BRM rabbit monoclonal (Cell Signaling Technology, 11966T, Lot# 5)
H3K4me1 rabbit monoclonal (Cell Signaling Technology, 5326T, Lot# 7)
H3K4me3 rabbit polyclonal (Cell Signaling, 9727S, Lot# 6)
H3K27ac rabbit polyclonal (Sigma -Aldrich, 07-360, Lot# 3935826)
MED1 rabbit polyclonal (Bethyl Laboratories, A300-793A-T, Lot# 11)
Rabbit IgG HRP-conjugated (Invitrogen, 31460, Lot# RB230194)
Mouse IgG HRP-conjugated (Invitrogen, 31430, Lot# QD216575)
Rabbit IgG Alexa 647-conjugateed (Invitrogen, 21245, Lot# 1660844)
Mouse IgG Alexa 800-conjugateed
Rabbit IgG Alexa 488-conjugateed

Validation

BRG1: Validated for IF, CUT&RUN, WB and IP by manufacture (https://www.abcam.com/en-us/products/primary-antibodies/brg1-antibody-epncir111a-ab110641), and for ChIP-seq in other study (PMID: 34446700). In this study, WB signal are disappeared after AU-15330 treatment, supporting antibody specificity.
HA-Tag (rabbit): Validated for WB, IP, IF and ChIP by manufacture (https://www.cellsignal.com/products/primary-antibodies/ha-tag-c29f4-rabbit-mab/3724?srsltid=AfmBOoqlmaMbX6xuW6Rn59VFSV89zLd5B6Gk6Ervl8gPdMCn-L_zMFLR), and for WB in other study (PMID: 33558760). In this study, CUT&RUN signal are disappeared after dTAG treatment, supporting antibody specificity.
HA-Tag (mouse): Validated for WB and IF by manufacture (https://www.cellsignal.com/products/primary-antibodies/ha-tag-6e2-mouse-mab/2367?srsltid=AfmBOorCUxE2zacjtsM-VdNVaDe2a6EbSDHPPiF8X4S6xwfBiHZSoaPl), and for WB in other study (PMID: 39747581)
ARID1A: Validated for WB and IF by manufacture (https://www.novusbio.com/products/arid1a-antibody-cl3595_nbp2-61623?srsltid=AfmBOopiaEZaLvlorrjaBkgETKIHfoi_p5nhIhNUp-dr5vPmu7qQDBab), and for IF in other study (PMID: 39013863)
PBRM1: Validated for IHC, IP and WB by manufacture (https://www.fortislife.com/products/primary-antibodies/rabbit-anti-pbrm1-antibody/BETHYL-A301-591), and for WB in other study (PMID: 34937944). In this study, WB signal are disappeared after AU-15330 treatment, supporting antibody specificity.
BRD9: Validated for WB and IP by manufacture (https://www.fortislife.com/products/primary-antibodies/rabbit-anti-brd9-antibody/BETHYL-A303-781), and for WB in other study (PMID: 32457312)
HDAC1: Validated for WB and IF by manufacture (https://www.novusbio.com/products/hdac1-antibody-cl0510_nbp2-52937?srsltid=AfmBOopt4ExBq3IBBcaRfcg0qhWpTpSKDwpRkq6Sx4KWEXTuqmMG413j)
SNF2H: Validated for IP, WB and IF by manufacture (https://www.abcam.com/en-us/products/primary-antibodies/snf2h-antibody-ab72499), and for WB in other study (PMID: 30996347)
Rhodamine-conjugated Tubulin: Validated for WB by manufacture (https://www.bio-rad.com/en-us/sku/12004165-hfab-rhodamine-anti-tubulin-primary-antibody-200-ul?ID=12004165), and other study (PMID: 37730997)
BRM: Validated for WB, IP, IF and ChIP by manufacture (https://www.cellsignal.com/products/primary-antibodies/brm-d9e8b-xp-rabbit-mab/11966?srsltid=AfmBOoqZdOajF695KLJ03dwnwvZqCJr4qmEYqefAh1rFgvaVIEi7494z), and for ChIP-seq in other study (PMID: 34446700). In this study, CUT&RUN and WB signals are disappeared in BRM-KO cells and AU-15330 treated cells, supporting antibody specificity.
H3K4me1: Validated for WB, IF, ChIP CUT&Tag, and CUT&RUN by manufacture (https://www.cellsignal.com/products/primary-antibodies/mono-methyl-histone-h3-lys4-d1a9-xp-rabbit-mab/5326?srsltid=AfmBOopcC2WSYuc_3WeyNYvWtAm2wTp7h20tYc3xXBiHHnRneSzkE0Es),and for CUT&Tag in other study (PMID: 39582024)
H3K4me3: Validated for WB and IF by manufacture (https://www.cellsignal.com/products/primary-antibodies/tri-methyl-histone-h3-lys4-antibody/9727?srsltid=AfmBOorFdwncUVJ4BZt-GA4kDvLpHiTRelgt1TUYkSXtVTi4VlwG3hRm), and for ChIP-seq in other study (PMID: 35668082)
H3K27ac: Validated for ChIP-seq and WB by manufacture (https://www.sigmaaldrich.com/US/en/product/mm/07360?srsltid=AfmBOopkSh7Onz1crwF-amvCtLQLs4T10iirV738zGsHG14Ud26-98lN), and for CUT&RUN in other study (PMID: 39747581)
MED1: Validated for IHC, IP and WB by manufacture (https://www.fortislife.com/products/primary-antibodies/rabbit-anti-med1-antibody/BETHYL-A300-793), and for ChIP-seq in other study (PMID: 26416749)
Rabbit IgG HRP-conjugated and Mouse IgG HRP-conjugated:Validated for WB in other study (PMID: 31209294)

Eukaryotic cell lines

Policy information about cell lines and Sex and Gender in Research

Cell line source(s)

Male mouse ES cell line of C57BL/6J background was established in this study. Drosophila S2 cells can be obtained from Thermo Fisher Scientific (R69007). BEAS-2B cells can be obtained from American Type Culture Collection (ATCC, CRL-3588)

Authentication

Authentication Genotype of cell lines was tested at the level of DNA sequence and protein.

Mycoplasma contamination

The ES cell lines and BEAS-2B cell line were tested negative for mycoplasma contamination, Drosophila S2 cells were not tested.

Commonly misidentified lines (See ICLAC register)	None

Animals and other research organisms

Policy information about studies involving animals; ARRIVE guidelines recommended for reporting animal research, and Sex and Gender in Research

Laboratory animals	Female (C57BL/6J, 8 week-old) were mated with Male (C57BL/6J, 10 week-old) and used to collect blastocyst stage embryos.
Wild animals	No wild animals were used in this study.
Reporting on sex	Female mouse was used for blastocyst collection and Male mouse was used for mating
Field-collected samples	No field-collected samples were used in this study.
Ethics oversight	All animal experiments were performed in accordance with the guidelines of the Institutional Animal Care and Use Committee at Harvard Medical School. All mice were kept under specific pathogen-free conditions within and environmental controlled for tempurature (20-22°C) and humidity (40–70%), and were subjected to a 12-hour light/dark cycle.

Note that full information on the approval of the study protocol must also be provided in the manuscript.

Plants

Seed stocks	N/A
Novel plant genotypes	N/A
Authentication	N/A
