# Supplementary material for: Precise modulation of BRG1 levels reveals features of mSWI/SNF dosage sensitivity
Source: Nat Genet. 2025 Aug 22;57(9):2250–63. doi: 10.1038/s41588-025-02305-z (PMC12425804; doi:10.1038/s41588-025-02305-z)
Supplement: Supplementary file 4 — Uncropped western blot and gel images for Figs. 1, 3 and 6 and Extended Data Figs. 1 and 8. [file 41588_2025_2305_MOESM4_ESM.pdf]

Fig. 1a

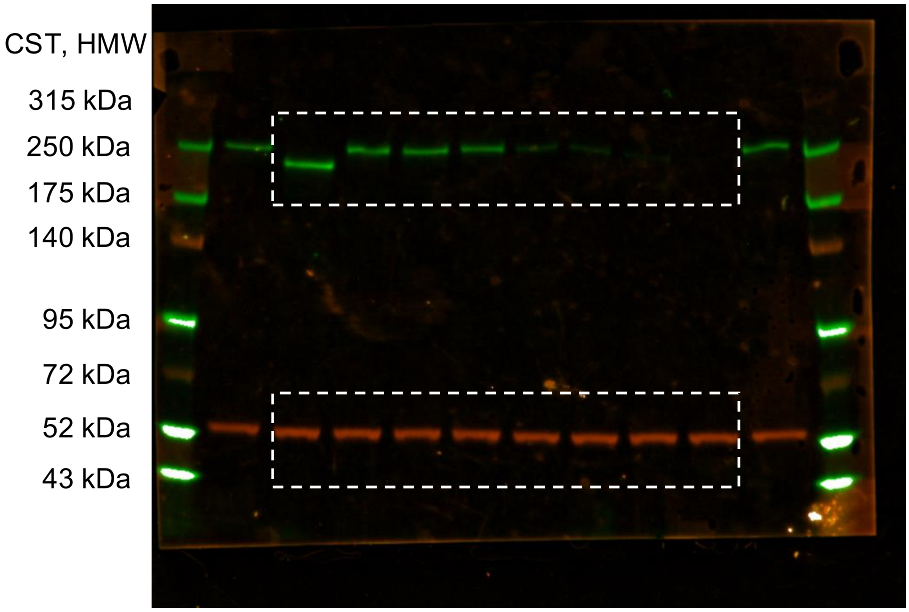

Green: BRG1

Red: β-Tubulin

Fig. 3d

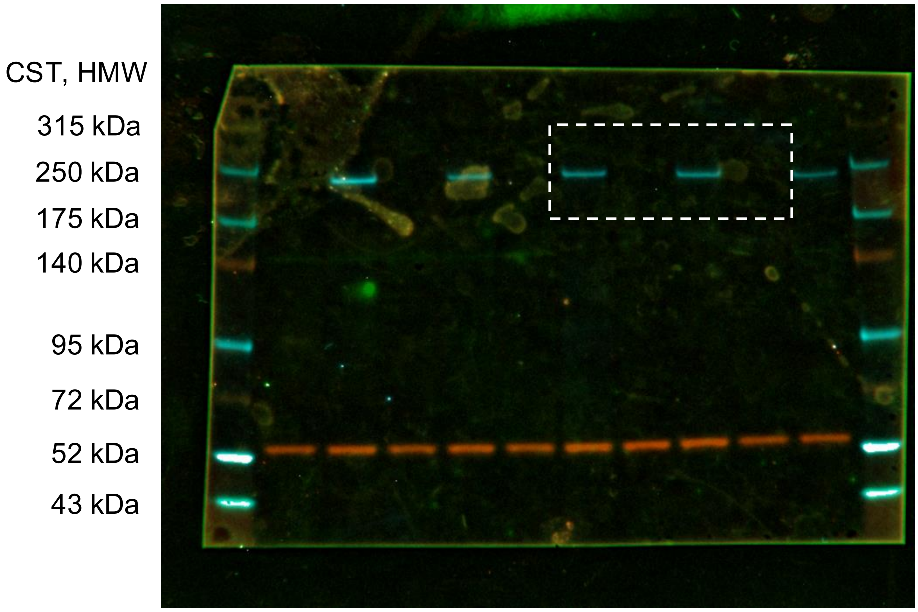

Cyan: BRG1

Red: β-Tubulin

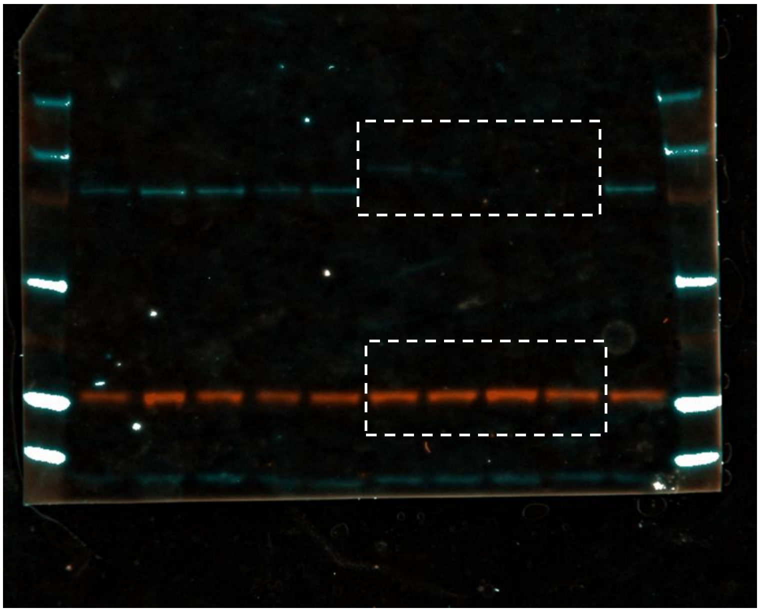

Cyan: SNF2H

Red: β-Tubulin

Fig. 6a

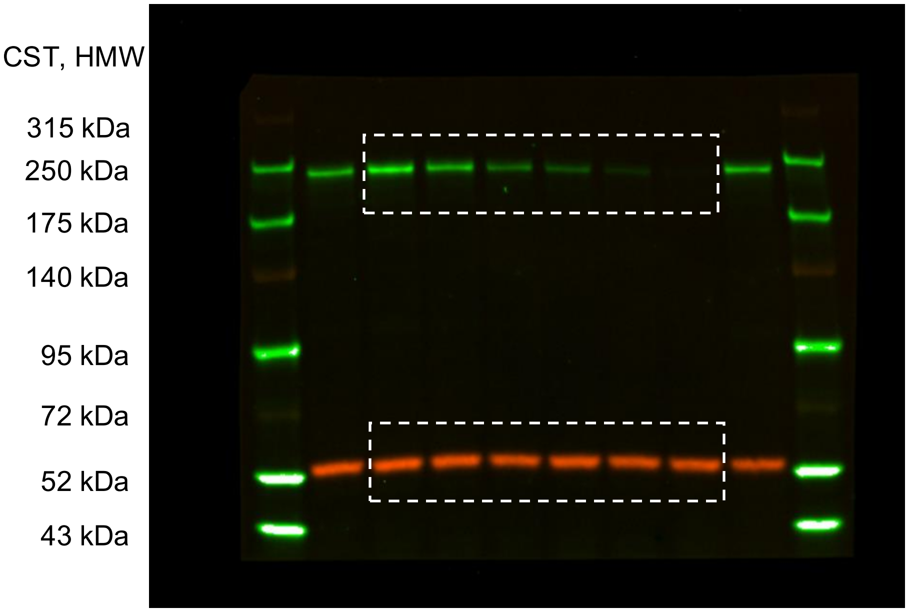

Green: BRG1

Red: β-Tubulin

Source Data for Extended Data Fig. 1d

CST, HMW
315 kDa
250 kDa
175 kDa
140 kDa
95 kDa
72 kDa
52 kDa
43 kDa

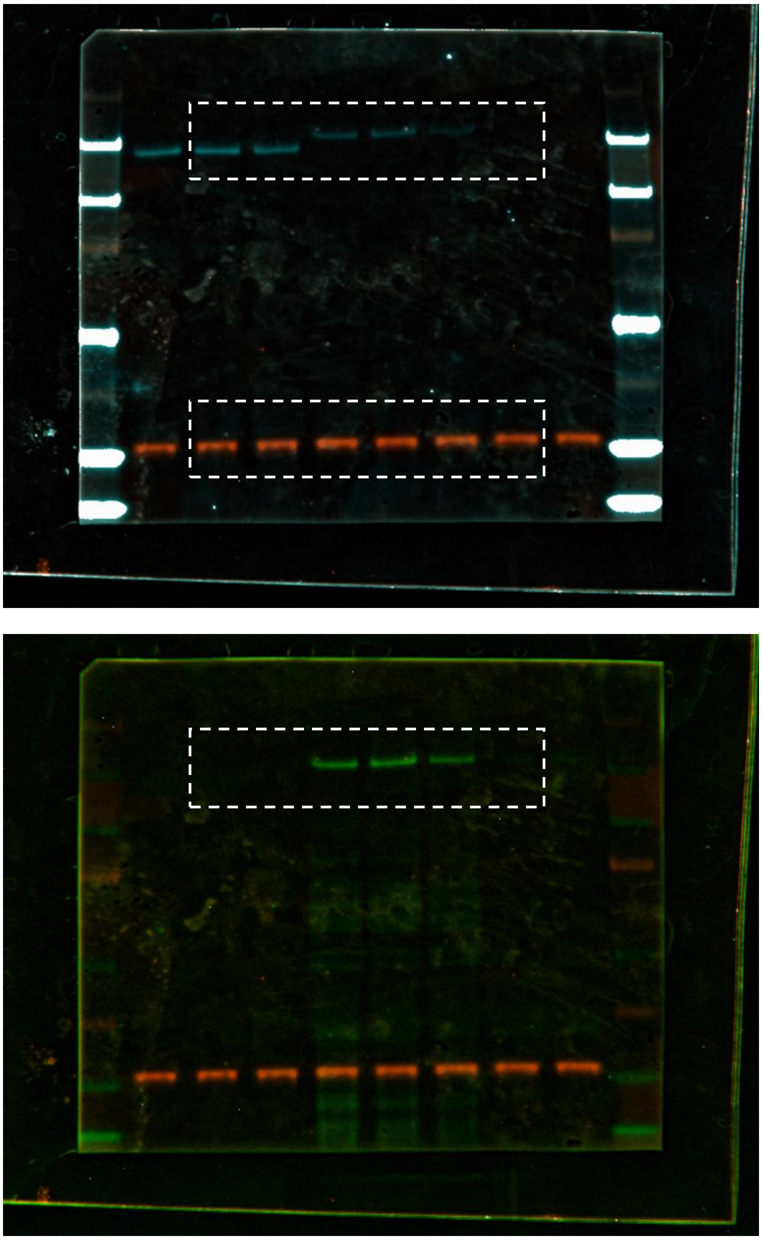

Cyan: BRG1

Green: HA-tag

Red: β-Tubulin

Source Data for Extended Data Fig. 1e

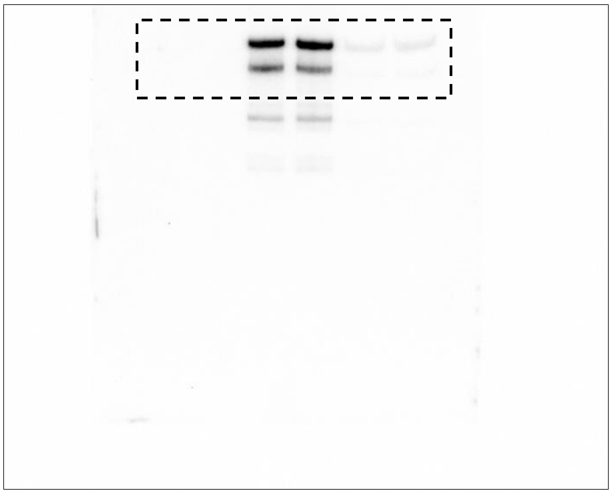

ARID1A

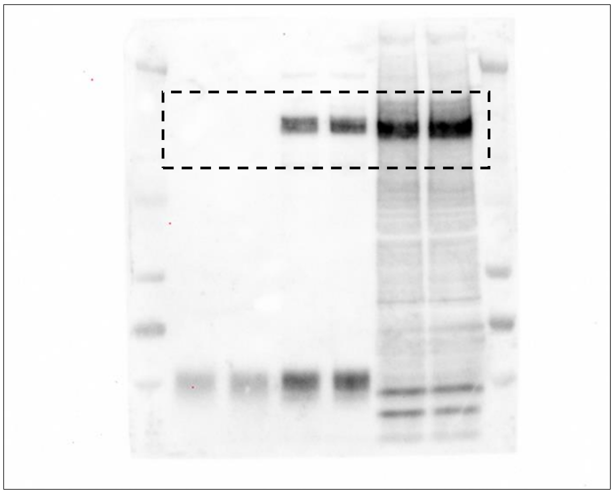

PBRM1

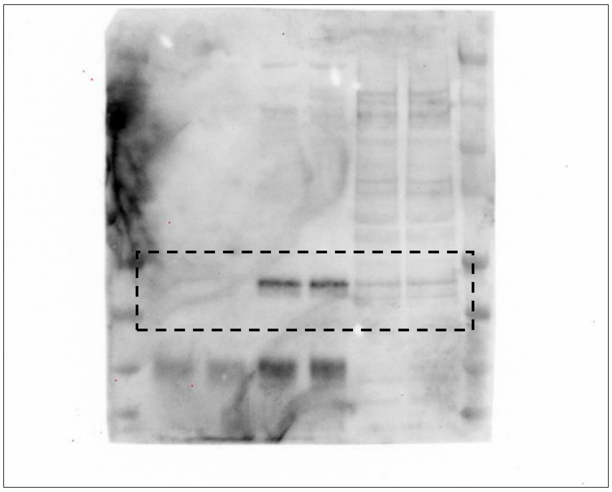

BRD9

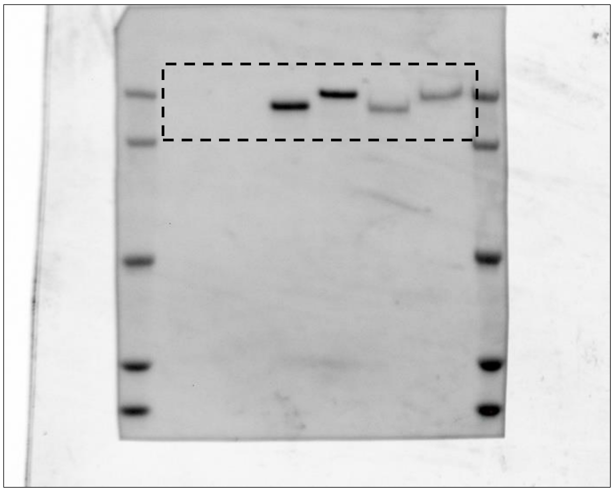

BRG1

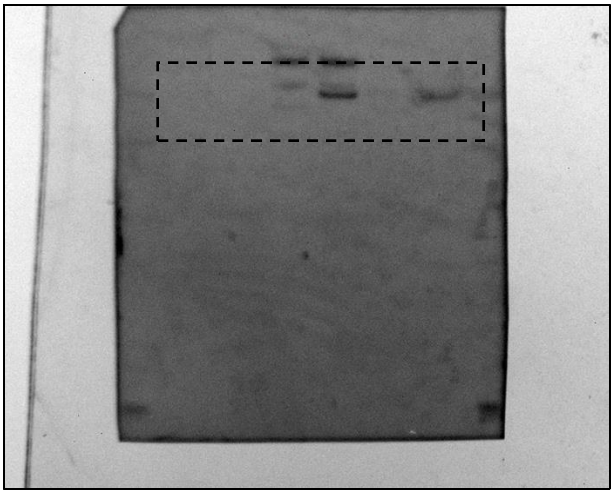

HA-tag

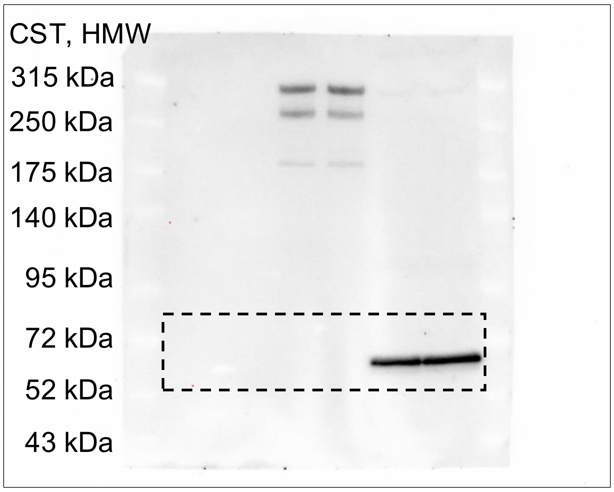

HDAC1

Source Data for Extended Data Fig. 8a

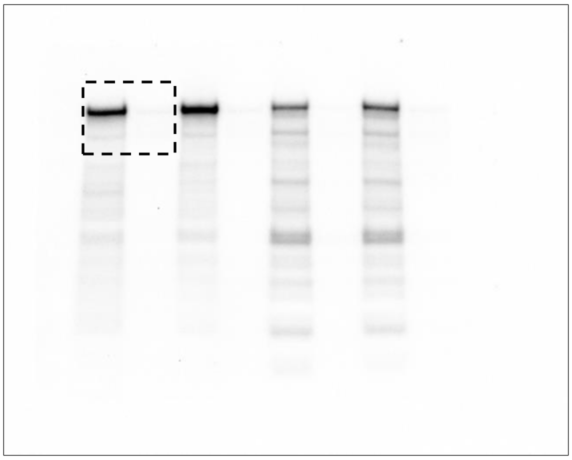

BRG1

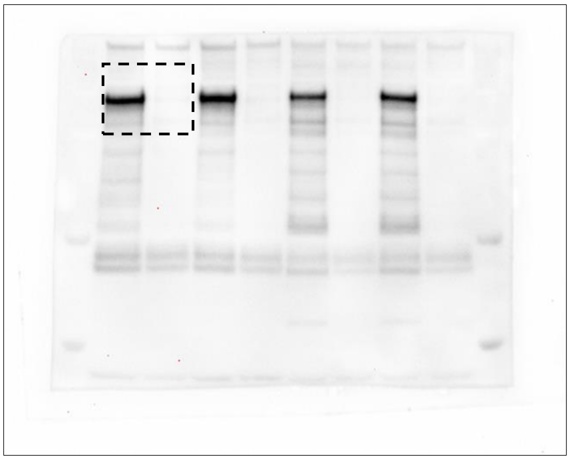

BRM

CST, HMW
315 kDa
250 kDa
175 kDa
140 kDa
95 kDa
72 kDa
52 kDa
43 kDa

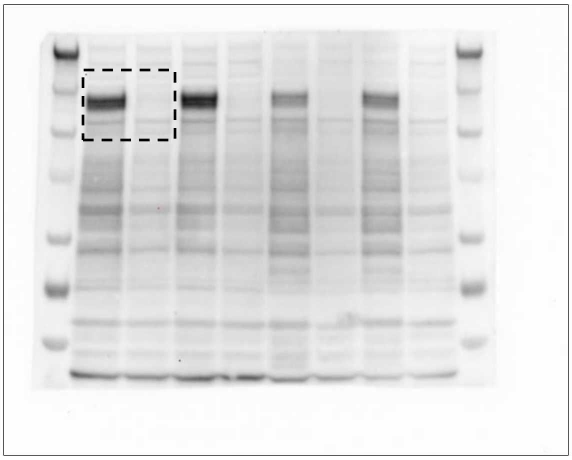

PBRM1

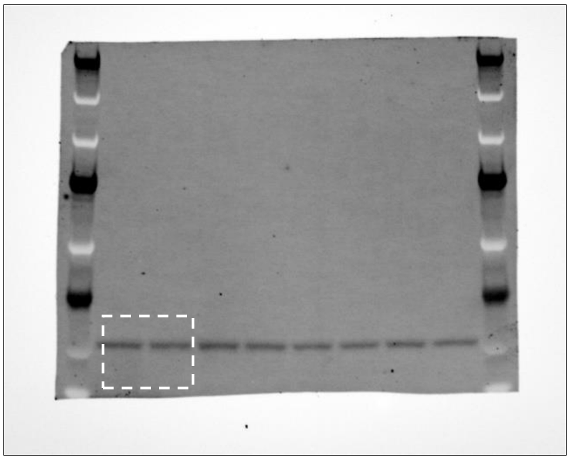

β-Tubulin

Source Data for Extended Data Fig. 8b

1 kb Plus
DNA Ladder
3,000bp
1,000 bp
500 bp
200 bp
100 bp

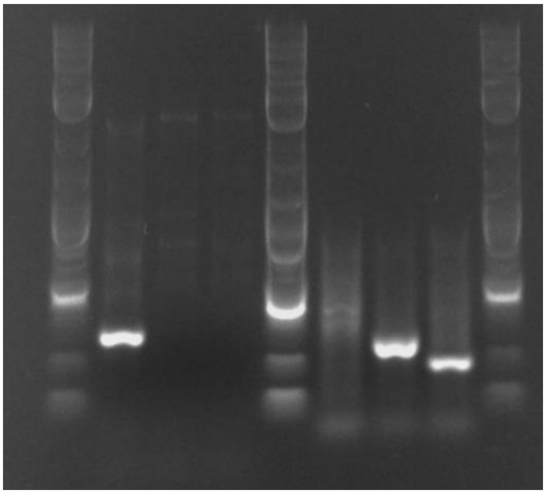

Source Data for Extended Data Fig. 8b

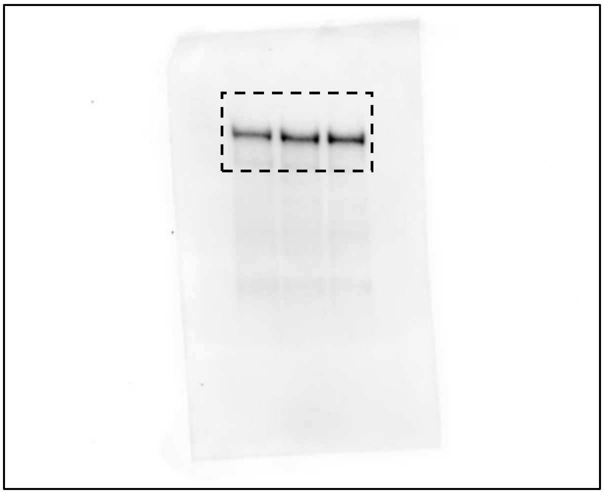
BRG1

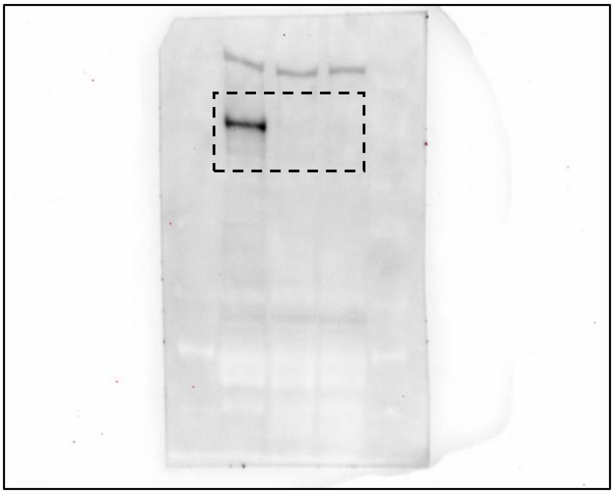
BRM

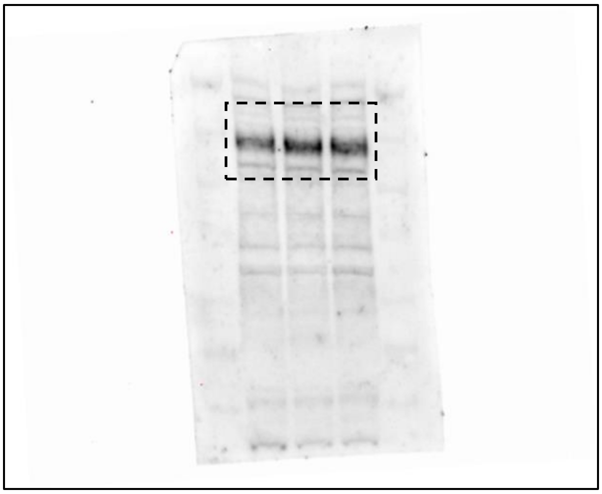
PBRM1

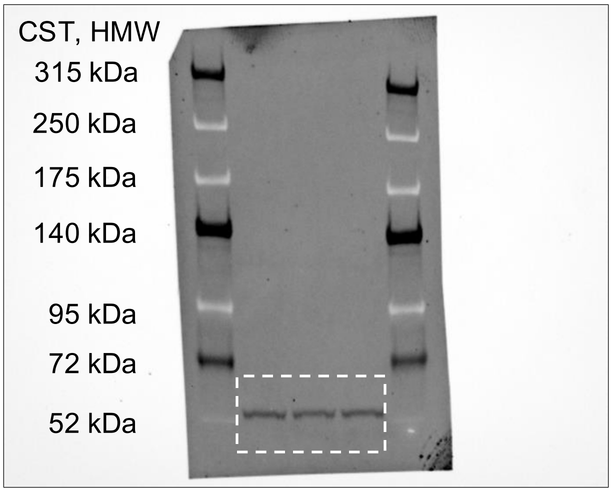

β-Tubulin

Source Data for Extended Data Fig. 8e

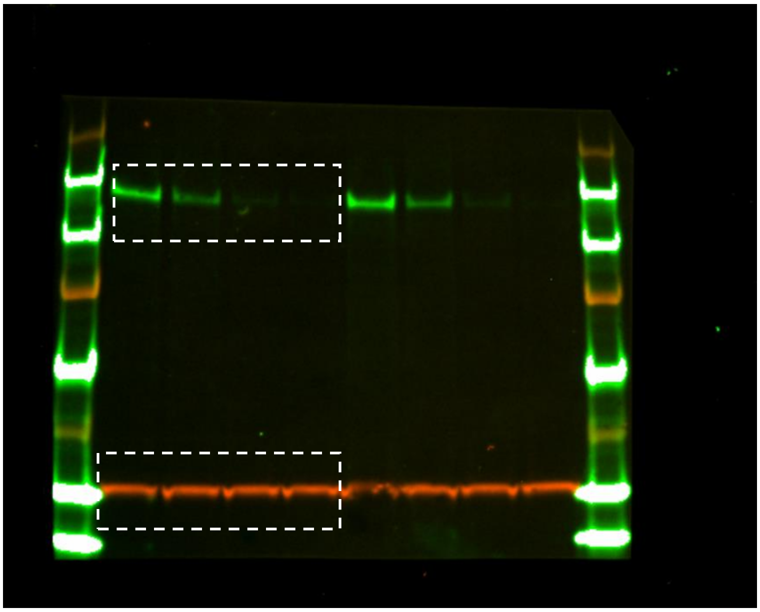

CST, HMW
315 kDa
250 kDa
175 kDa
140 kDa
95 kDa
72 kDa
52 kDa
43 kDa

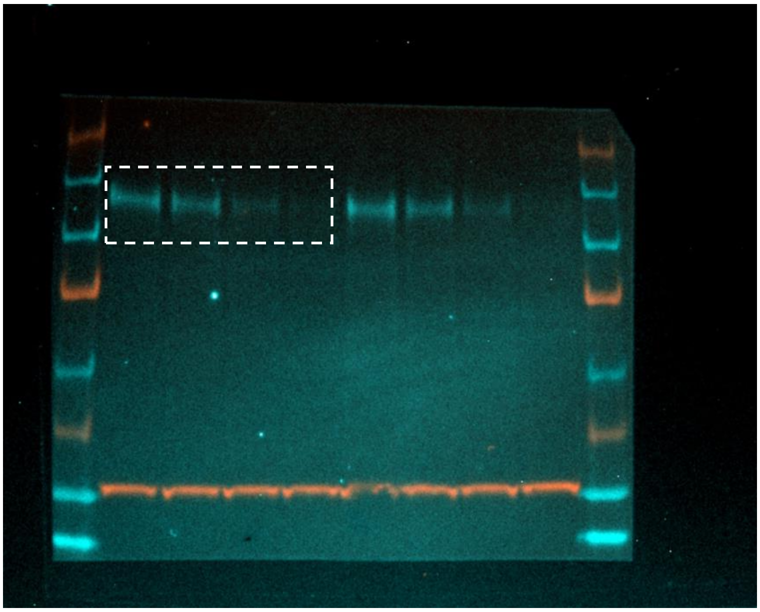

Green: BRG1

Cyan: PBRM1

Red: β-Tubulin

Source Data for Extended Data Fig. 8h

CST, HMW
315 kDa
250 kDa
175 kDa
140 kDa
95 kDa
72 kDa
52 kDa
43 kDa

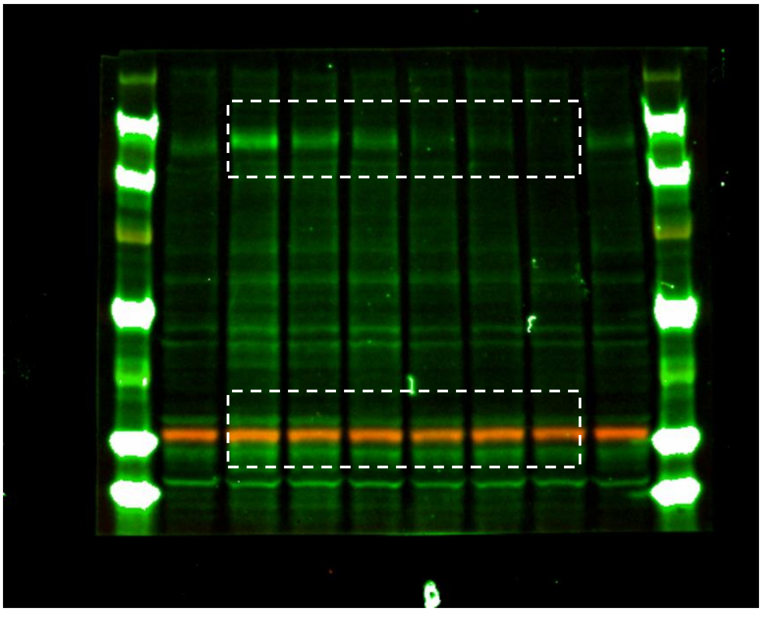

Green: PBRM1

Red: β-Tubulin
